# Understanding histone deacetylation in Huntington’s disease

**DOI:** 10.18632/oncotarget.13924

**Published:** 2016-12-12

**Authors:** Luis Miguel Valor

**Affiliations:** ^1^ Unidad de Investigación, Hospital Universitario Puerta del Mar, Cádiz, Spain

**Keywords:** epigenetics, transcription, neurodegenerative, Neuroscience

Huntington’s disease (HD) is a neurodegenerative disorder that affects 5 to 10 people per 100,000 inhabitants and it is caused by an aberrant expansion of trinucleotide CAG repeats (>36) in the exon 1 of the huntingtin gene. As a consequence of this mutation, a complex symptomatology of cognitive decline, motor impairment, personality changes and other alterations is usually manifested in mid adulthood until the final death of the patient. Despite the establishment of the monogenic causative nature of HD twenty years ago, there is still no effective therapy.

In the early 2000s a promising ameliorative strategy was conceived after the observation of deficiencies in histone acetylation levels in several HD models, and the detection of an enzyme involved in this modification, the acetyltransferase CREB-binding protein (CBP), in mutant huntingtin aggregates [1]. Because acetylation of histone tails is an epigenetic modification associated with active genes and postulated to mediate chromatin relaxation and facilitate transcription, this pathological histone deacetylation attracted soon the researchers’ attention as a plausible cause for the prominent transcriptional dysregulation observed in HD, which affects relevant genes for brain functioning and survival. Almost instantly to these findings, treatment with HDAC inhibitors (HDACis) was reported to restore histone deacetylation in parallel to the amelioration of certain pathological traits. Since then, an increasing number of covalent modifications of both histones and DNA have been added to the repertoire of molecular alterations in HD, accompanied by reports describing the benefits of epigenetic-based drugs in both cellular and animal models, and by clinical trials in patients [1-3]. Actually, the relevance of epigenetics is not restricted to HD but also well established in aging and other neurodegenerative disorders, including more prevalent conditions such as Alzheimer’s disease and Parkinson’s disease. However, the rather simplistic view of the proposed role of epigenetics in neuropathology has been challenged in recent years. For example, epigenetics as a causal force of transcription is far from being well understood, and HDACis as paradigms of epigenetic-related drugs have multiple actions beyond histones (see [1, 2, 4, 5] for further discussion). Therefore, the molecular basis of amelioration remains to be fully dilucidated.

In 2013 we published the most complete genomic landscape of histone acetylation in the context of mutant huntingtin expression, namely the ChIP-seq analysis of acetylated lysines 9 and 14 of histone H3 and acetylated lysine 12 of histone H4 in the hippocampus of the transgenic N171-82Q strain [6]. This work represents a good example of the surprisingly limited correlation between epigenetic impairment and transcriptional dysregulation, not evidenced by single gene studies but revealed using genome-wide approaches and extended to other epigenetic modifications [1, 4, 7]. In the absence of bulk level changes, there was a global trend towards histone hypoacetylation that was, at first sight, in concordance with the predominance of downregulation in gene expression over upregulation [6]. However, only a small subset of genes showed a reliable convergence of differential expression and differential acetylation of histone H3 at transcription start sites (TSS) [6]. In agreement with this observation, the genome-wide efforts aimed at the identification of HDACi targets in HD models have yielded candidates that are weakly related with the transcriptional dysregulation program or that need to be replicated [1, 4]. In any case, this small subset contained potential relevant genes for synaptic and neuronal functions that also exhibited similar alterations in the striatum and associated cortex of another HD model, the R6/1 strain [8], suggesting the existence of a consistent transcriptional and epigenetic signature across HD mouse models that can be potentially restored by using pharmacological approaches.

In this follow-up study [8] we also introduced the concept of susceptibility to transcriptional dysregulation that may help to better reconcile alterations at the epigenetic and transcriptional level. In other words, histone H3 deacetylation of TSS may mark genes that become affected at the gene expression level only under specific but still unknown circumstances. In this article we provided some examples of genes effectively hypoacetylated at their TSS that showed specific altered patterns of gene expression depending on the mouse model (e.g., *Fos*), brain area (e.g., *Igfbp5*) or stage of the disease (e.g., *Camk1g* and *Rasl11b*). In fact, whereas only 12% of the hypoacetylated genes in the hipocampus of N171-82Q was also transcriptionally altered in this brain area and animal model, this percentage was increased to 41% when also considering the genes transcriptionally altered in other datasets generated from diverse HD scenarios (i.e., N171-82Q cerebellum, R6/1 striatum, R6/2 cortex and striatum, and caudate nucleus of early-grade patients) [8]. Taking into account that this meta-analysis can be considered as preliminary, it is expected that further analytical improvements will not only strength the relationship between pathological epigenetics and transcription but also will enhance our understanding of the transcriptional actions of epigenetic restorative approaches. In addition, these results suggested that dysregulation of histone acetylation in HD must converge with spatial and/or temporal specific factors (i.e., other gene expression regulators such as transcription factors, chromatin-remodeling proteins, non-coding RNAs, etc.) to produce effective transcriptional alterations [4]. The identification of these regulatory partners will be key to properly define the molecular mechanisms and genuine targets of epigenetic-based ameliorative strategies.
